# Treatment of Deciduous Teeth

**Published:** 1880-09

**Authors:** J. F. Adams

**Affiliations:** Worcester, Mass.


### TREATMENT OF DECIDUOUS TEETH.

BY DR. J. F. ADAMS, OF WORCESTER, MASS.

An encouraging sign of progress in the practice of our specialty is to be found in the increasing attention given to the treatment of deciduous teeth. Twenty, or even fifteen, years ago, there were few dentists who had sufficient sense of the importance of these teeth to induce them to attempt any thorough or systematic treatment for their preservation; and there were few parents who would not have laughed at the notion of filling their children's first teeth. Thanks to the efforts of good men in the profession, to the influence of dental societies, dental colleges and the dental magazines, a change for the better is taking place in the profession; the importance of these teeth is more generally appreciated among us, and, as a consequence, the people are beginning to believe that they are designed to serve an important purpose, the fulfillment of which demands their preservation.

There is still, however, a great field for missionary work in this direction; there are still hundreds of people who never even heard of filling deciduous teeth, and there are hundreds more who, while recognizing the value of our operations upon their own teeth, are doubtful of the advantage of bestowing the same care upon those of their children.

Such persons are surprised when we tell them that these teeth are designed to remain from five to ten years, and that, if left to themselves, they will, in a majority of cases, become diseased in three or five years, creating a source of frequent irritation and suffering to the child, and that, if allowed to remain in this condition, or if they are extracted, the mastication of food, imperfect enough under the most favorable circumstances, becomes impossible. It goes to the stomach with almost no preparation, and the result is indigestion, which is the cause of most of the minor ills to which young children are subject.

You tell them that this constant disturbance of the digestive functions can not but have its effect in lowering the general tone of the system, diminishing its power of resisting disease, so that when the child is attacked with scarlet fever or measles its chances of recovery are correspondingly lessened. You explain, also, that the premature extraction of these teeth is a frequent source of irregularity in the permanent set, which will have to be corrected by a tedious and expensive process of regulating, or the victim must suffer from an unsightly deformity as long as the teeth last. These arguments are convincing, and if the questioners are intelligent they will thank you for the instruction, and will profit by it.

Many dentists shrink from volunteering such information to their patients, fearing that their motive may be misconstrued, and that they may be suspected of having an ax of their own to grind. Such a consideration ought not to have any weight with a man who labors for the best good of his patrons. For the family dentist, especially, there is no excuse for allowing his patients to remain in ignorance in regard to this subject. It is not only a professional privilege to give advice in such matters, but it is a duty which he owes to them. It is almost wholly from conversation with the dentist that the public gain what knowledge they have in regard to their teeth, and he should be to them what his title implies—a teacher.

To deal with little patients requires a good degree of tact. We must lay aside our dignity, for the time, and come down to their level, in order to secure their confidence and good will, which are very necessary to the accomplishment of our object. A child may be frightened into opening his mouth to have a tooth extracted, but he will not stay scared long enough to have a dozen cavities filled; so we must adopt the course which will be best in the long run.

To be sure, it sometimes requires the possession of most of the Christian virtues in an eminent degree, with a patience of which Job might well have been proud, but dentists are expected to possess all these qualities.

In operating for very young children, after filling the crown cavities, it is a good practice, in many cases, before treating any of the proximal cavities, to make all the separations that are required.

This is done in anticipation that, before we are through with him, our patient will become *im*-patient, and refuse to have the work completed.

If a child's fears have not been excited by injudicious words of encouragement from his parents, or of warning from his sisters and his other relatives, he comes for the first sitting with perfect willingness. The novelty of the situation is sufficient to hold his attention for an hour or so, and, if he has been properly handled, he will look forward with pleasant anticipations to another visit. He comes, and sits through another appointment, and perhaps a third, and still says he has had a good time; but, very likely, when we next see him, the little wretch has changed his views on the subject entirely. It was all well enough, he thinks, for once or twice, but the thing is getting monotonous. He prefers some other kind of amusement, and no amount of coaxing will induce him to alter his determination that tooth-filling is a bore. You may try the "mild, but firm," dodge with him, but it doesn't work. He knows when he has had enough.

Now I believe the best possible thing to do with a child in that frame of mind is to let him alone for three or four months, when we may find that "Barkis is willin'." In the meantime, if we have succeeded in separating his teeth so as to expose the decayed surfaces to the friction of mastication, we have retarded the progress of decay, so that no serious harm has resulted. There are but few cases where this cannot be accomplished, as, for some reason, young children seldom have the nervous shrinking from the file that we find in our adult patients. When, however, as is sometimes the case, we meet with one who utterly refuses to submit to the treatment, we may, when all other means have failed, resort to the use of ether. It is neither a difficult nor a dangerous thing to do for a young child; and I think the importance of the object is sufficient to warrant such a course.

I believe in making wide spaces, and generally use for the purpose a three-cornered file, which gives just the desired angle. Extreme caution is necessary if the disk is used.

Whatever may be said against Prof. Arthur's method of separating the permanent teeth, there certainly can be no objection to the practice when applied to deciduous teeth. They are admirably adapted, by their shape, for this mode of treatment, being always large at the neck. Hence the space, once gained, is always maintained, and the teeth are accessible on all sides. The wide angle of separation brings the proximal cavities to the surface, so to speak, rendering them almost as simple as those in the crowns. There is no occasion for making the double V-shaped space in separating these teeth, the better way being to file in a direct line from the buccal to the palatal or lingual side, and carry the file down to the gum.

In treating the incisors and mesial surfaces of the cuspids, the cutting may be done from the palatal side, according to the Arthur plan, and even if the decay be not entirely removed, it will be so arrested that nothing further will be needed—for the incisors, that is—except an occasional labial filling, as they will probably last as long as they are required.

Of the manner of filling, little need be said. Perhaps a word of caution to inexperienced operators in regard to excavating may not be out of place. It must be remembered that the pulp is larger in proportion to the size of the tooth than is that of the permanent tooth, and great caution is necessary to avoid too near approach to it. It takes on inflammation at the slightest provocation, and will die every time if exposed, and abscess is pretty sure to follow. In case of the exposure of the pulp, the safest way is to destroy it with arsenic, used very cautiously, and allowed to remain but a short time. Remove the dead pulp, and drill from beneath the gum into the pulp chamber, and in filling leave the roots and lower portion of the chamber open, to allow the escape of any gases through the opening which has been made.

Many operators recommend tin foil as superior to all other materials for filling deciduous teeth; but are its advantages over amalgam sufficient to compensate for the greater time required for its introduction? Time is an important element in these operations; what we do must be done quickly. We all know that amalgam serves well in crown cavities; and if the teeth are separated in the manner described, proximal cavities become, in effect, crown cavities, and amalgam answers all the requirements. For buccal and labial cavities, there is nothing better than gutta-percha.

The most important of the deciduous teeth, in their relation to second dentition, are the cuspids and the second molars. The former, being the last in the natural order of shedding, are the sentinels which must remain at their posts to prevent their successors from being crowded out of the arch; and the latter, which guard the outposts, can not with safety be relieved from duty, at least until the six-year molars are firmly in place, otherwise the latter will encroach upon the territory belonging to the bicuspids. Hence, if we are only able to save a part of the teeth, these two classes should receive our chief attention.

Another point in reference to second dentition: When the development of the permanent incisors, upper and lower, takes place symmetrically—that is, when it takes place in both jaws nearly at the same time—the lower incisors usually come regularly into their places; but when, as is often the case, the development of the lower incisors is considerably in advance of that of the upper, the expansion necessary for their natural eruption can not take place, owing to the interlocking of the temporary cuspids. The consequence is, the former, especially the laterals, make their appearance inside the arch, or are turned on their axes, presenting edgewise.

There is a very simple operation for the prevention of this irregularity, which was first brought to notice by Dr. J. T. Codman, and which I have practiced with perfect success in a good many cases. It is simply to grind the points of the cuspids, either upper or lower, or both, so as to unlock them. This gives the lower cuspids freedom to move in a lateral direction, and a short time after the operation they will be found standing out laterally beyond the upper ones, being pushed out by the incoming incisors, which will take their natural positions in the jaw.—*Trans. Conn. Valley D. S.*

